# Building a better blueprint for bolting

**DOI:** 10.1093/plcell/koae240

**Published:** 2024-08-23

**Authors:** Nora Flynn

**Affiliations:** Assistant Features Editor, The Plant Cell, American Society of Plant Biologists; Department of Botany and Plant Sciences, University of California Riverside, CA 92507, USA

Development occurs through both gradual changes and rapid transformations. In annual plants like Arabidopsis, an initial vegetative phase swiftly switches to a later reproductive phase, leading to bolting, or the growth of a flower stalk. Bolting is accompanied by leaf senescence as the plant focuses on setting seed. This reproductive transition requires large-scale transcriptional reprogramming that remains poorly understood but has significant implications for yield and nutrient quality in crops (Havé et al. 2017). Through single-plant-omics, **Ethan Redmond and colleagues (Redmond et al. 2024)** explore how gene expression changes during bolting, simplifying an immensely complex, shifting transcriptional landscape into orderly events.

Studying the transcriptional change that precedes bolting presents many challenges. For example, even in nearly isogenic populations, plants may not uniformly bolt on the same day of development. On top of that, the rapid transcriptional shift during bolting likely involves numerous, sequential changes that would require sampling at many timepoints to visualize. To overcome these difficulties, the authors developed a single-plant-omics approach to reveal how gene expression is reorganized during the switch to reproduction. Individual libraries for RNA-sequencing were made for approximately 70 *Arabidopsis thaliana* (ecotype Ws) plants that had been sown together and grown for 20 days (see Fig. A and B). At this time, bolting status was also classified, with roughly one-third of the plants visibly beginning to bolt.

**Figure.**
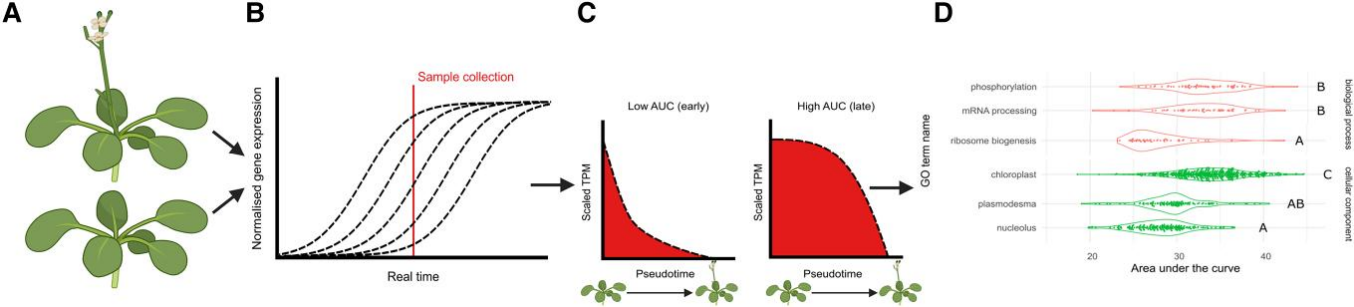
Revealing sequential transcriptional changes during bolting. **A)** Bolted and unbolted plants collected after 20 days. **B)** RNA sequencing of developmentally asynchronous plants. **C)** Calculating AUC for transcripts that decrease over pseudotime during bolting. Low AUC indicates a transcriptional decrease that occurs earlier in pseudotime, while high AUC indicates a transcriptional decrease that occurs later. **D)** Comparison of GO terms by AUC values. Adapted from Redmond et al. (2024), Figure 3. Created with BioRender.com.

Hierarchical clustering of the gene expression data showed two transcriptionally unique groups that were correlated with bolted or unbolted plants. Between these two groups of plants, 55% of the transcriptome was differentially expressed, including upregulation of programmed cell death genes in the senescing, bolted plants. However, with such a colossal transcriptional switch, narrowing down specific processes during bolting from the vast networks involved would be difficult.

To unravel the genes with the biggest impact during bolting, the authors used the asynchrony of the plants’ developmental stage to their advantage (see Fig.). Although all the plant samples were the same chronological age, they were not the same biological age because only some had bolted. By ordering the plants by biological age, also known as pseudotime, the trajectory of transcriptional changes from unbolted to bolted plants could be revealed. First, genes were classified as “increasing” or “decreasing” during bolting to track their transcript levels over pseudotime. For example, transcripts classified as “decreasing” should have high levels in unbolted, biologically younger plants and lower levels over pseudotime, though the relative timing of decreased expression would vary by gene.

To visualize the relative timing of the transcriptional change, the authors compared the area under the curve (AUC) values from the relationship between transcript level and pseudotime (see Fig. C), where higher AUC values correspond to stronger relationships. As an example, if a “decreasing” gene shows an early drop in transcript level (such as before bolting), its AUC value will be low compared with another gene that maintains its transcript level for some time before decreasing. Therefore, for “decreasing” genes, low AUC would correspond to earlier timing of the change compared with high AUC. By exploring the gene ontology (GO) terms associated with different AUC values, the authors could order transcriptional events, revealing that senescence-related processes, like reduced ribosome biogenesis, occur even before visible bolting (see Fig. D). Further, establishment of pseudotime opened the door to explore bolting-associated gene regulatory networks, which included members of the SQUAMOSA PROMOTER BINDING PROTEIN-LIKE (SPL) family that were previously reported to be involved in the transition to the reproductive phase (Xu et al. 2016).

Not only did the authors investigate rapid, bolting-associated changes, they also investigated genes that influence gradual developmental changes like biomass and leaf size, including several AGAMOUS-LIKE MADS-box transcription factors previously identified as predictors of yield (De Meyer et al. 2023). Overall, the impressive analytical methods by Redmond and colleagues strategically explore both rapid and gradual developmental factors, illuminating major candidates and sequential events to begin to build a blueprint of the transcriptional changes during bolting.
